# Hydrate formation at extreme conditions

**DOI:** 10.1107/S2053229622011731

**Published:** 2023-01-01

**Authors:** Simon Parsons

**Affiliations:** a EaStCHEM School of Chemistry and Centre for Science at Extreme Conditions, The University of Edinburgh, King’s Buildings, West Mains Road, Edinburgh EH9 3FJ, United Kingdom

**Keywords:** salt hydrate, high pressure, intercalation, commentary, potassium chloride

## Abstract

The article of Yamashita *et al.* [*Acta Cryst.* (2022), C**78**, 749–754] on water intercalation into the B1 structure of KCl under high pressure illustrates the ability of *in situ* crystal growth to by-pass kinetic barriers between phases, demonstrating the importance of this approach in phase discovery.

A search of the Inorganic Crystal Structure Database for hydrates of the group I binary halides shows that the most extensive series is formed by lithium, for which mono-, di- and trihydrates are known for the chloride, bromide and iodide. The most highly hydrated species are the penta­hydrates formed by LiCl and LiBr (Sohr *et al.*, 2018 ▸). Below lithium, the hydrate chemistry drops off quickly. Sodium chloride, bromide and iodide form only dihydrates, and amongst the heavier elements, only KF·2H_2_O (Preisinger *et al.*, 1994 ▸), KF·4H_2_O (Beurskens & Jeffrey, 1964 ▸) and RbF·H_2_O (Troyanov, 2005 ▸) are known. This series of materials contrasts with the halides of the more highly charged group II metals, which have an extensive hydrate chemistry, including some very highly hydrated examples, such as MgCl_2_·12H_2_O (Sasvari & Jeffrey, 1966 ▸). The propensity towards hydrate formation by lithium can be regarded as an example of a diagonal relationship in the periodic table.

Inter­est in hydrates of simple salts stems from their importance as possible mineral phases on icy planets and moons. While the majority of work has been carried out at ambient pressure, characterizing the behaviour of hydrates at high pressure, yielding data such as bulk moduli, as well as new and perturbed crystal structures, is important for developing geophysical models of extraterrestrial environments (Grindrod *et al.*, 2010 ▸; Maynard-Casely, 2017 ▸).

In the December issue of *Acta Crystallographica Section C*, Yamashita *et al.* (2022 ▸) report the crystal structure of a new monohydrate of KCl, obtained at 2.23 GPa by *in situ* crystal growth from a saturated aqueous solution contained in a diamond anvil cell (Komatsu *et al.*, 2011 ▸). The crystal grows together with high-pressure phases of ice, and the authors controlled the crystallization conditions to ensure that the concomitant phase was ice-VII, which is cubic with *a* ∼ 3.3 Å, rather than the more complex ice-VI, which led to greater contamination of the diffraction pattern. The novel design of the diamond anvil cell (which is described in more detail in the supporting information) had an unusually large opening angle to optimize data completeness.

KCl itself crystallizes in the NaCl structure (also called ‘B1’) under ambient conditions, but in the CsCl structure (‘B2’) above 2 GPa, the higher coordination numbers of the ions enable a more efficient packing even if the bond distances are slightly longer, an example of what Kleber has termed the *pressure–distance paradox* (Müller, 2006 ▸). The structure of KCl·H_2_O can be considered to have some of the characteristics of both phases. The first impression is of the NaCl structure which has become distorted to accommodate the water, but the potassium is eight-coordinate, as in the CsCl-like phase. The positions of the H atoms, which are very important for understanding the crystal structures of hydrates, were located from the diffraction data and confirmed using periodic density functional theory (DFT) calculations.

Although chemically simple, the salt hydrates are very sensitive to external conditions, with complex energy landscapes containing multiple viable phases. However, transformations between solid phases are frequently kinetically hindered, and KCl·H_2_O is not accessible by, for example, compressing solid KCl in the presence of water. The article of Yamashita *et al.* illustrates the ability of *in situ* crystal growth to bypass kinetic barriers between phases, demonstrating the importance of this approach in phase discovery (Oswald *et al.*, 2008 ▸; Ridout & Probert, 2013 ▸; Moggach & Oswald, 2020 ▸).
